# Cholinergic Differentiation of Human iPSCs Reveals Early APOE4-Driven Dysregulation of Neuronal Markers, Synaptogenesis and Inflammatory Responses

**DOI:** 10.3390/cells15121057

**Published:** 2026-06-09

**Authors:** Nele Johanne Czaniera, Wiebke Schulten, Katja Nowak, Diana Pschik, Jonas Joneleit, Barbara Kaltschmidt, Christian Kaltschmidt

**Affiliations:** 1 Department of Cell Biology, University of Bielefeld, 33615 Bielefeld, Germany; wiebke.schulten@uni-bielefeld.de (W.S.); katja.nowak@uni-bielefeld.de (K.N.); diana.pschik@uni-bielefeld.de (D.P.); c.kaltschmidt@uni-bielefeld.de (C.K.); 2 Forschungsverbund BioMedizin Bielefeld, Ostwestfalen-Lippe (OWL) (FBMB e.V.), 33611 Bielefeld, Germany; jonas.joneleit@uni-bielefeld.de (J.J.); barbara.kaltschmidt@uni-bielefeld.de (B.K.); 3 Institute for Laboratory and Transfusion Medicine, Heart and Diabetes Centre NRW, Ruhr-University Bochum, 32545 Bad Oeynhausen, Germany; 4 Department of Molecular Neurobiology, University of Bielefeld, 33615 Bielefeld, Germany

**Keywords:** Alzheimer’s disease, *APOE*, *APOE3*, *APOE4*, cholinergic neurons, hiPSCs-derived neurons, neuronal differentiation, TNF-α, neuroinflammation

## Abstract

Alzheimer’s disease (AD) is a progressive neurodegenerative disorder characterized by progressive memory impairment and cognitive decline. The *APOE4* allele represents one of the most prominent genetic risk factors. In this study, we investigated the impact of *APOE4* on the cholinergic neuronal development and on the neuronal inflammatory response to TNF-α stimulation. To address this, human induced pluripotent stem cells (hiPSCs) carrying a homozygous *APOE4* genotype and an isogenic *APOE3* control were differentiated into cholinergic-like induced neurons (iNs) by *LHX8* overexpression. *APOE4* was associated with accelerated early neuronal differentiation, as reflected by earlier downregulation of the progenitor marker Nestin. However, delayed expression of synaptophysin indicated impaired synaptic maturation. Functionally, *APOE3* iNs exhibited a robust but temporally regulated response to TNF-α, whereas *APOE4* iNs were characterized by a delayed yet sustained induction of inflammatory signaling. Moreover, *APOE4* iNs displayed an enhanced stress-associated transcriptional response at early differentiation stages. Collectively, these findings suggest that *APOE4* influences both neuronal development and the timing and persistence of inflammatory responses, potentially predisposing cholinergic neurons to later dysfunction in AD.

## 1. Introduction

Despite extensive research, Alzheimer’s disease (AD) remains a major health and societal challenge [1,2]. As the most common form of dementia, AD is characterized by the accumulation of amyloid-β (Aβ)-containing plaques and neurofibrillary tangles composed of hyperphosphorylated tau, which are associated with progressive neuronal loss [3,4,5,6]. However, early detection remains challenging, often resulting in diagnoses at later stages when the disease has already substantially progressed [5]. AD is typically classified into two subtypes: early-onset (EOAD) and late-onset (LOAD). While LOAD is defined by an onset after the age of 65 and is influenced by multiple risk factors, EOAD is strongly linked to inherited mutations [7]. Nevertheless, genetic factors also contribute substantially to the risk of developing LOAD [6,8]. One of the most important genes in this context is the apolipoprotein E (*APOE*) gene, particularly the *APOE4* allele.

*APOE4* is associated with an up to 12-fold increased risk of developing AD [9]. In contrast, the *APOE3* variant is generally considered neutral with respect to AD risk and differs from *APOE4* by a single amino acid. While ApoE3 carries a cysteine at position 112, ApoE4 contains an arginine at this position [10,11]. This structural difference contributes to functional alterations, including changes in lipid transport and a shift towards amyloid-β–related pathways [12,13,14]. Additionally, *APOE4* has been shown to exacerbate tau pathology and increase the level of pro-inflammatory cytokines [15,16,17]. Notably, these differences are not restricted to mature neurons but can already be observed in vitro at much earlier developmental stages, such as in human induced pluripotent stem cells (hiPSCs) [18].

These early alterations are reflected during neuronal differentiation, where *APOE4* has been reported to accelerate neuronal development, although there is also evidence for delayed progression, suggesting an overall dysregulation [19,20,21,22]. Furthermore, *APOE4* has been associated with an increased number of synapses. However, these synapses exhibit functional impairments, indicating synaptic dysfunction [19,23]. Nevertheless, many of the underlying mechanisms remain poorly understood.

Given that memory loss is one of the hallmark symptoms of AD, it is of particular interest to investigate the effects of *APOE4* during the differentiation of cholinergic neurons, which play a central role in learning and memory [24]. Cholinergic neurons utilize acetylcholine (ACh) as their primary neurotransmitter, which acts as a key regulator of these cognitive processes [25]. Moreover, cholinergic neurons represent a major therapeutic target through the inhibition of acetylcholinesterase (AChE). As this enzyme is responsible for the cleavage of acetylcholine (ACh) into acetate and choline, its inhibition leads to increased ACh levels [26]. Currently, several drugs are available as treatment options, such as tacrine, donepezil, galantamine, or huperzine A. However, these therapies primarily alleviate symptoms rather than targeting the underlying disease mechanisms [27,28].

In vitro cell-based models provide a powerful platform to investigate fundamental differences between *APOE3* and *APOE4*. One commonly used approach involves the direct differentiation of somatic cells or iPSCs through the overexpression of specific transcription factors (TFs), enabling the generation of defined neuronal subtypes described as induced neurons (iN) [29]. LHX8, a LIM-homeobox transcription factor, has been identified as a critical regulator of cholinergic neuron development in mice and rats [30,31,32]. Studies in human cells have also shown that LHX8 regulates the expression of key cholinergic markers, including choline acetyltransferase (ChAT) and the vesicular acetylcholine transporter (VAChT), which play critical roles in the synthesis and release of ACh [33].

To investigate the effects of APOE4 on the development of cholinergic-like neurons, we established a rapid differentiation method. Specifically, doxycycline-inducible expression of *LHX8* was introduced into iPSCs to drive cholinergic lineage specification. To model disease-relevant stress conditions, cells were exposed to tumor necrosis factor alpha (TNF-α), a pro-inflammatory cytokine, as elevated levels of TNF-α have been detected in the serum of Alzheimer’s patients [34,35]. Further reports indicated that TNF-α contributes to the regulation of neuronal degeneration [36]. In addition, elevated TNF-α levels have been associated with reduced functional connectivity in the brain, an effect that appears to be further amplified in the presence of the *APOE4* allele, suggesting a brain environment that is more susceptible to inflammation [37].

We investigated the effects of *APOE4* on cholinergic neuronal differentiation and stress responses using a doxycycline-inducible, LHX8-based hiPSCs model. Our data show that *APOE4* adversely affects the expression of synaptic markers, particularly within the cholinergic lineage. In addition, we identified genotype-dependent differences in the inflammatory response that persisted across developmental stages, indicating that both early-stage and more mature neurons are differentially impacted by *APOE4*.

## 2. Materials and Methods

### 2.1. Cell Culture and Neuronal Differentiation

The homozygous hiPSCs used were obtained from The Jackson Laboratory (Bar Harbor, ME, USA). All details of the cell lines are provided at https://www.jax.org/jax-mice-and-services/ipsc/cells-collection/JIPSC001150 (accessed on 1 June 2026). These include the cell line JIPSC001150 and its revertant JIPSC001162, which have previously been characterized in detail by Schulten et al. [18]. As this study was based on one *APOE4* hiPSC line and its corresponding isogenic *APOE3* revertant control, biological replication at the level of independent donors or independent hiPSC lines was not applicable. Experimental reproducibility was assessed using independent differentiation experiments and technical replicates, as indicated in the respective figure legends. Both transduced and non-transduced hiPSCs were cultured in 6-well plates (Sarstedt, Nümbrecht, Germany) coated with 5 µg/mL vitronectin (Thermo Fisher Scientific, Waltham, MA, USA). Cells were maintained in Essential 8 Flex medium (E8 Flex medium; Thermo Fisher Scientific, Waltham, MA, USA) at 37 °C and 5% CO_2_.

For neuronal differentiation, cells were passaged on day -1 at approximately 80% confluency. Either 6-well plates or μ-Slide 8-well chambers (Ibidi, Gräfelfing, Germany) were used, both coated with 50× Geltrex (Thermo Fisher Scientific, Waltham, MA, USA) according to the manufacturer’s instructions. On day 0, the medium was supplemented with 0.1 µg/mL doxycycline (Sigma-Aldrich, St. Louis, MO, USA), which was maintained throughout the entire differentiation period. Neuronal differentiation medium consisted of Neurobasal medium (Thermo Fisher Scientific, Waltham, MA, USA) supplemented with 2× B-27 Supplement (Thermo Fisher Scientific, Waltham, MA, USA), 2 mM L-glutamine (Sigma-Aldrich, St. Louis, MO, USA) and 1% penicillin/streptomycin. The medium was changed daily until day 5, as summarized in Table 1.

On day 5, 50 ng/mL Brain-Derived Neurotrophic Factor (BDNF; PeproTech, Cranbury, NJ, USA) and 50 ng/mL Nerve Growth Factor (NGF; PeproTech, Cranbury, NJ, USA) were added to the differentiation medium. From this point onward, the medium was partially changed (1/3 of the total volume) every 2–3 days.

### 2.2. Lentiviral Production and Transduction

HEK293FT cells (Invitrogen, Carlsbad, CA, USA) were used for production of lentiviral particles and were cultured in DMEM high glucose (PAN-Biotech, Aidenbach, Germany) supplemented with 2 mM L-glutamine (Sigma-Aldrich, St. Louis, MO, USA) and 10% fetal calf serum (FCS; Sigma-Aldrich, St. Louis, MO, USA). One day prior to transfection, 4 × 10^6^ cells were seeded per TC100 dish (Sarstedt AG and Co., Nümbrecht, Germany). The medium was replaced 2 h prior to transfection. For transfection, the plasmids FUW-M2-rtTA, which was a gift from Rudolf Jaenisch (Addgene-Plasmid Nr. 20342, [38]) and pTet-O-LHX8-T2A-PuroR, which was a gift from Charles Gersbach (Addgene plasmid # 162350, [29]) were used. The Lentivirus system was a gift from David Baltimore. Preparation and packaging (plasmid Δ8.91) was done with pseudotyping with the vesicular stomatitis virus glycoprotein (VSVG) as described below [39]. Each transfer vector was co-transfected using the calcium phosphate precipitation method. After 24 h, the medium was replaced, and after an additional 29 h, the supernatant containing viral particles was collected. Following this, the supernatant was filtered using a 0.45 µm PES filter and concentrated using Lenti-X Concentrator (Takara Bio, Saint-Germain-en-Laye, France) according to the manufacturer’s instructions. Then, hiPSCs were transduced with viral particles in the presence of polybrene and 0.5 µM ROCK inhibitor thiazovivin (Sigma-Aldrich, St. Louis, MO, USA). After three additional passages, single-cell cloning was performed to isolate transduced cell lines. The analysis was based on one cell line per genotype. Successful transduction was confirmed by RT-PCR following 2 days of doxycycline induction, selecting for cells expressing LHX8.

### 2.3. Immunocytochemistry

Cells were fixed with 4% paraformaldehyde for 10 min at room temperature (RT), followed by three washing steps with 1× phosphate-buffered saline (PBS; PAN-Biotech, Aidenbach, Germany). For blocking and permeabilization, cells were incubated for 1 h at RT in a solution containing 1× PBS, 0.02% Triton X-100 (Sigma-Aldrich, St. Louis, MO, USA), and 5% goat serum (Dianova, Hamburg, Germany). Subsequently, cells were incubated with primary antibodies (listed in Table 2) for 1 h at RT.

After three washing steps with 1 × PBS, cells were incubated with secondary antibodies at a dilution of 1:300, including Alexa Fluor 555 (goat anti-rabbit) or Alexa Fluor 488 (goat anti-mouse; both Thermo Fisher Scientific, Waltham, MA, USA). For nuclear visualization, mounting medium containing DAPI (Ibidi, Gräfelfing, Germany) was applied.

Image acquisition was performed using a confocal laser scanning microscope (Leica STELLARIS 8 FALCON; Leica Microsystems GmbH, Wetzlar, Germany). Image processing was carried out using the OMERO.figure web module [40] and CorelDRAW Graphics Suite 2023 (Corel Corporation, Ottawa, ON, Canada). The analysis was performed using ImageJ 1.54p (National Institutes of Health, Bethesda, MD, USA), and statistical analysis as well as heat-map visualization were performed using GraphPad Prism (version 8.3.0). For this, three microscopic images from each cell line were analyzed. The mean fluorescence was calculated as follows:

Mean fluorescence = (Integrated density − (image area × mean control background))/cell count(1)

For this calculation, the entire microscopic image was selected as the region of interest.

### 2.4. TNF-α Treatment

TNF-α treatment was performed on days 4 and 14 of neuronal differentiation. For this purpose, cells were seeded in 6-well plates (Sarstedt, Nümbrecht, Germany) as described above. The culture medium was supplemented with 10 ng/mL TNF-α (PeproTech, Cranbury, NJ, USA), and cells were treated either for 1 h or 4 h. Following treatment, cells were washed with 1× PBS (PAN-Biotech, Aidenbach, Germany) and collected for RNA isolation as described below.

### 2.5. RT-PCR and RT-qPCR

To assess gene expression and verify successful transduction, total RNA was isolated from the cells using the NucleoSpin^®^ RNA Kit (Macherey-Nagel, Düren, Germany). Subsequently, cDNA was synthesized using the First Strand cDNA Synthesis Kit (Thermo Fisher Scientific, Waltham, MA, USA), following the manufacturer’s protocol.

For RT-PCR, Taq DNA polymerase (New England Biolabs (NEB), Frankfurt am Main, Germany) was used. PCR products were analyzed by agarose gel electrophoresis (2%) and stained with Midori Green (NIPPON, Düren, Germany).

The RT-qPCR measurements were performed using the CFX96 Real-Time PCR Detection System (Bio-Rad Laboratories, Hercules, CA, USA). Reactions were prepared in a total volume of 10 µL using Luna Universal qPCR Master Mix (NEB, Frankfurt am Main, Germany) and were run in technical triplicates. Basal gene expression levels were normalized to the housekeeping genes *GAPDH* and *RPLP0*. Relative gene expression was analyzed using the ΔΔCq method. Cq values were first normalized to the housekeeping genes *GAPDH* and *RPLP0* to obtain ΔCq values. For time-course comparisons under untreated conditions, fold changes were calculated relative to *APOE3* iNs at day 4, as indicated in the respective figure legends. For TNF-α treatment experiments, fold changes were calculated relative to the respective untreated control of the same genotype and differentiation stage. The corresponding ΔCq values normalized to the housekeeping genes, prior to fold-change calculation, are provided in the Supplementary Material for all RT-qPCR analyses shown in Tables S3–S6. Data analysis was performed using Bio-Rad CFX Maestro software (version 4.1.2433.1219) and GraphPad Prism (version 8.3.0). All primers used in this study are listed in Table S1.

## 3. Results

### 3.1. LHX8 Transduced hiPSCs Maintain Pluripotency Marker Expression and Colony Morphology

**Figure 1:**
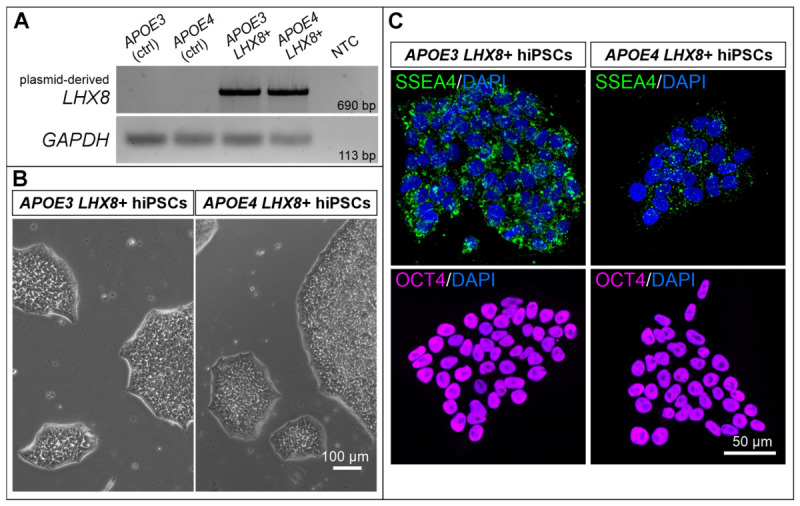
*LHX8* transduced hiPSCs retain pluripotency. (**A**) Comparison of transduced and as control untransduced hiPSC lines with either the *APOE3* or *APOE4* genotype. Plasmid-derived *LHX8* expression was detected exclusively in transduced cell lines. No-template control (NTC) samples showed no amplification. (**B**) Without doxycycline induction, transduced hiPSCs maintained their typical colony morphology. (**C**) Both transduced cell lines express the pluripotency markers SSEA4 and OCT4.

Successful *LHX8* transduction was confirmed using RT-PCR. Following 2 days of doxycycline induction, transduced *APOE3* and *APOE4* hiPSCs exhibited detectable expression of *LHX8* (Figure 1A). Notably, without doxycycline induction, hiPSCs continued to maintain typical colony morphology (Figure 1B). To assess whether the cells had lost their pluripotent state due to stress or the transduction process, immunocytochemistry (ICC) was performed for pluripotency markers (Figure 1C). Both the transcription factor OCT4 and the surface marker SSEA4 were detected in *APOE3* and *APOE4 LHX8*-positive hiPSCs, indicating that the cells had not undergone spontaneous differentiation as a result of transduction.

### 3.2. LHX8 Expression Drives Morphological Changes in hiPSCs Toward a Clustered Neuronal Phenotype

**Figure 2:**
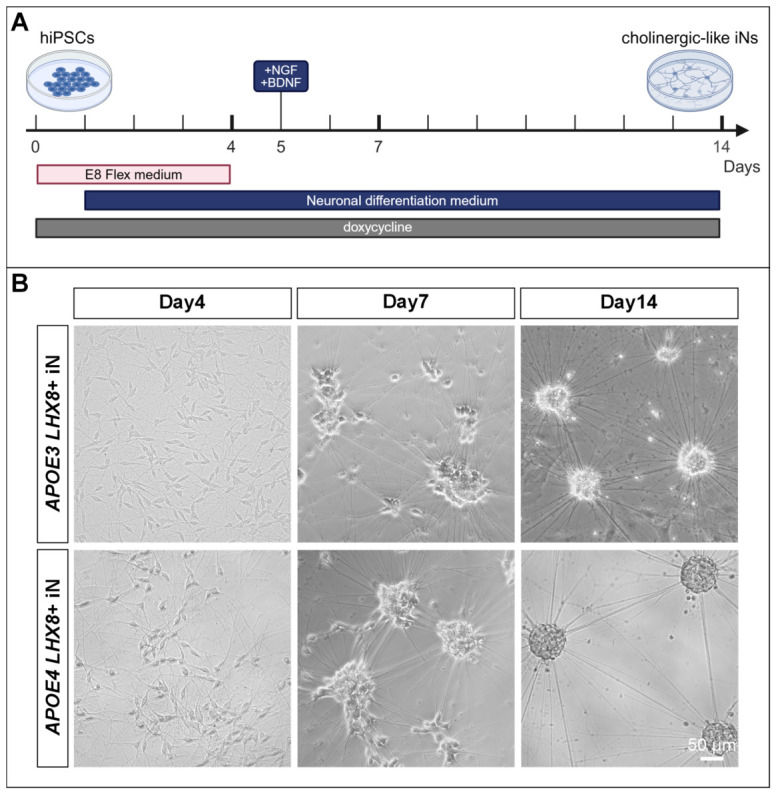
Morphological changes during *LHX8*-induced differentiation. (**A**) Schematic overview of the differentiation protocol. The iPSC medium (E8 Flex) was gradually replaced with neuronal differentiation medium. Doxycycline was continuously present throughout the whole time. On day 5, BDNF and NGF were added to the neuronal differentiation medium. Further analyses of iNs were performed on days 4, 7, and 14. (**B**) Representative microscopic images of iNs with either the *APOE3* or *APOE4* genotype. Cells progressively aggregated over time, forming more compact, cluster-like structures by day 14.

Following transduction, hiPSCs were seeded for neuronal differentiation. A gradual and slight medium transition from iPSC medium to neuronal differentiation medium was applied. On day 5, the neurotrophic factors NGF and BDNF were added to support neuronal growth and maturation (Figure 2A). After doxycycline induction, *LHX8* was expressed, leading to distinct morphological changes (Figure 2B). At an early stage (day 4), iNs already exhibited initial neurite-like projections. However, they remained largely isolated, with individual cells distributed rather than organized in clusters. By day 7, iNs progressively formed tighter aggregates, with cells becoming more closely arranged and giving rise to radial neurite outgrowth. This resulted in the formation of a network-like morphology. At this stage, no obvious genotype-dependent differences were observable based on morphology alone. By day 14, subtle morphological differences emerged: *APOE4* iNs appeared more densely packed and were characterized by more compact, spheroid-like neuronal aggregates with prominent neurite fasciculation, whereas *APOE3* iNs also formed aggregates but appeared less tightly organized and comparatively more loosely arranged.

### 3.3. Protein Expression Confirms Neuronal Differentiation and Reveals Differences in Synaptic Marker Levels Between APOE3 and APOE4 iNs

**Figure 3:**
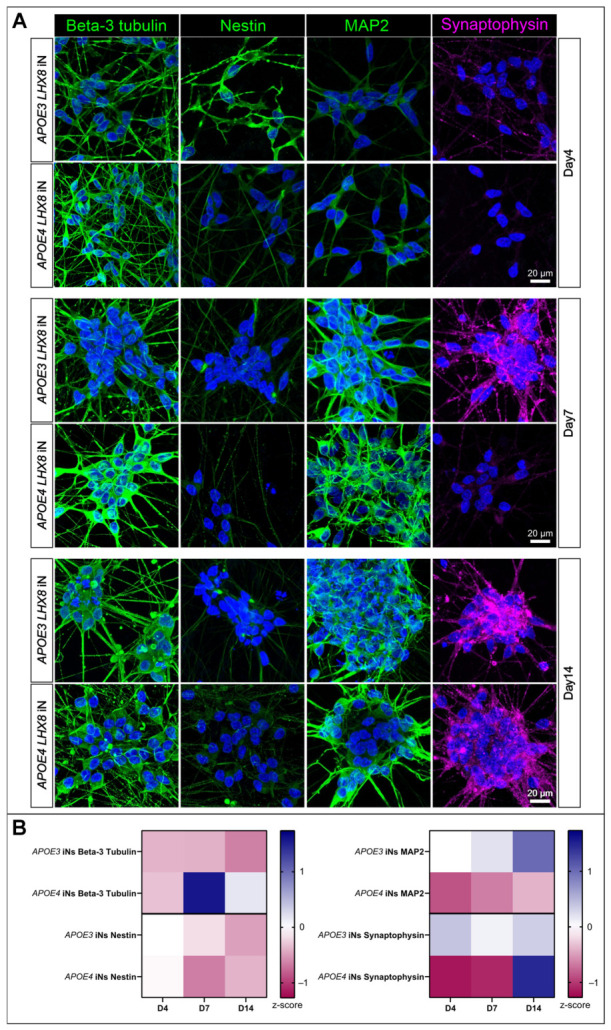
*LHX8* overexpression in hiPSCs induces the expression of multiple neuronal markers. (**A**) Comparative analysis of *APOE3* and *APOE4 LHX8*-positive iNs at day 4, 7 and 14. The protein markers Beta-3 tubulin, Nestin, MAP2 and synaptophysin were detected by ICC. Cell nuclei were counterstained with DAPI (blue). (**B**) Relative fluorescence intensities of Beta-3 tubulin, Nestin, MAP2, and Synaptophysin in *APOE3* and *APOE4* iNs at day 4, day 7, and day 14 of differentiation are shown as z-scores. Blue indicates higher and pink lower relative fluorescence intensity. Quantification was based on ICC staining, with three microscopic images analyzed per condition from one cell line. Mean fluorescence intensity was calculated from integrated density corrected for background and normalized to cell count.

To gain further insight into neuronal characterization, different proteins associated with neuronal identity were analyzed by immunocytochemistry (Figure 3A, Table S2). Beta-3 tubulin, a pan-neuronal structural marker, demonstrates that the typical network-like cellular morphology becomes increasingly interconnected over time, indicating progressive neuronal differentiation [41]. In addition, it confirms that the cells have differentiated toward a neuronal lineage. Nestin, a marker of neuronal progenitor cells, was slightly elevated in *APOE3* iNs at day 4, whereas it was already downregulated in *APOE4* iNs [42]. In *APOE3* iNs, Nestin expression decreased by day 7 and was thereafter only weakly expressed, reaching levels comparable to *APOE4* iNs. Consistent with other TF-mediated differentiation, positive staining for the neuronal marker MAP2 was already detectable from day 4 onwards [29,43]. MAP2 expression appeared comparable between *APOE3* and *APOE4* iNs at all time points. More noticeable differences were observed for the presynaptic marker synaptophysin [44]. While synaptophysin-positive cells were already detectable in *APOE3* iNs at day 4, *APOE4* iNs showed visibly reduced or nearly absent synaptophysin staining at this time point, despite similar to slightly higher MAP2 expression. By day 14, synaptophysin staining in *APOE4* iNs increased and visually approached levels observed in *APOE3* iNs. To further summarize and visualize genotype- and time-dependent changes across the analyzed neuronal markers, the corresponding immunocytochemistry signal intensities were additionally displayed as a heat map (Figure 3B). This overview supports the marker-specific observations, showing an early reduction in Nestin in *APOE4* iNs and a delayed increase in synaptophysin compared with *APOE3* iNs. In summary, *APOE4* iNs exhibit slightly accelerated differentiation, but show delayed pre-synaptic maturation.

### 3.4. Cholinergic Marker Expression Suggests Impaired Synaptic Maturation in APOE4 iNs

**Figure 4:**
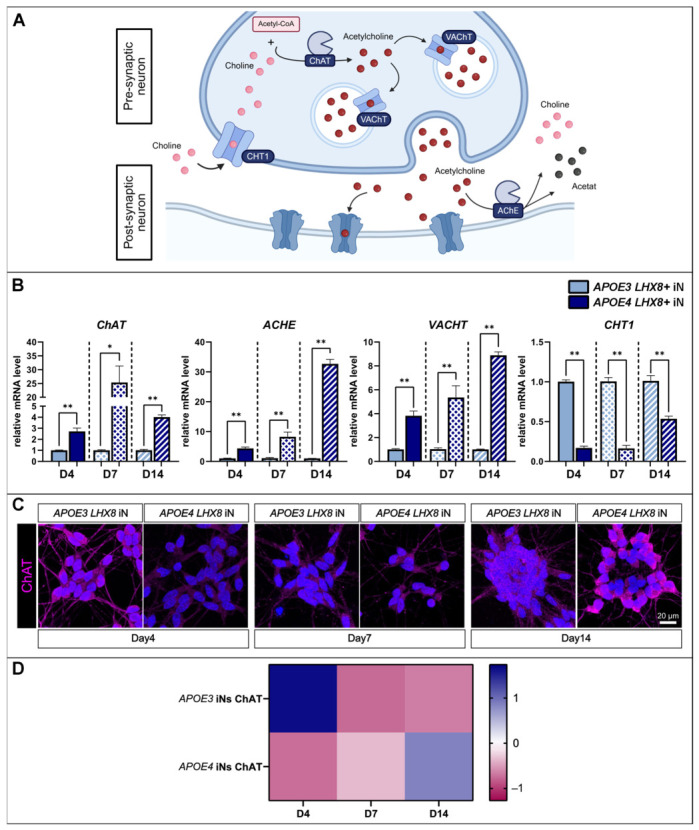
Detection of cholinergic marker expression in *LHX8*-induced iNs. (**A**) Schematic representation of a cholinergic pre- and postsynapse. The choline transporter CHT1 mediates the uptake of choline into the presynaptic terminal, where ChAT catalyzes it into ACh. VAChT packages ACh into synaptic vesicles, which are subsequently released into the synaptic cleft. ACh then binds to different receptors at the postsynapse. AChE degrades ACh into choline and acetate. (**B**) The mRNA levels of presynaptic markers were quantified by RT–qPCR and compared across differentiation time points. Data are presented as mean ± SEM. All samples were normalized to housekeeping genes. The mRNA level of *APOE4* iNs were analyzed relative to *APOE3* iNs from the same differentiation day using the Mann–Whitney test for each gene at the respective time point. Statistical significance was defined as * *p* < 0.05, ** *p* < 0.01. (**C**) Protein expression of ChAT was analyzed by ICC at days 4, 7, and 14, comparing *APOE3* and *APOE4* iNs. The cell nuclei were stained with DAPI (blue). (**D**) ChAT fluorescence intensity was quantified in *APOE3* and *APOE4* iNs at day 4, day 7, and day 14 of differentiation and visualized as z-scores. Blue represents increased relative ChAT fluorescence intensity, while pink indicates reduced relative fluorescence intensity. Quantification was based on ICC images, with three microscopic fields analyzed per condition from one cell line. Mean fluorescence intensity was calculated using background-corrected integrated density normalized to cell count.

Key cholinergic markers at the presynapse include ChAT, VAChT, AChE, and CHT1 (Figure 4A). These proteins are essential for the synthesis, transport, and degradation of ACh, a central neurotransmitter in cholinergic signaling [28,45,46]. Initially, mRNA of all analyzed genes could be detected at day 4, day 7, and day 14. Notably, at mRNA level, all genes except *CHT1* were more strongly expressed in *APOE4* iNs compared to *APOE3* iNs (Figure 4B, Mean ΔCq values in Table S3). By comparison, *CHT1* expression was significantly increased in *APOE3* iNs at all analyzed time points. At protein level, ChAT, one of the most characteristic markers of cholinergic neurons, was detected at all time points in both genotypes (Figure 4C) [47]. However, *APOE3* iNs displayed higher ChAT fluorescence levels at day 4, followed by a gradual decrease over time, whereas *APOE4* iNs showed an increase in ChAT fluorescence signal during differentiation (Figure 4D). In contrast, the glutamatergic marker vGLUT2 remained undetectable, supporting the cholinergic identity of the cells (Figure S1).

### 3.5. APOE4 iNs Exhibit Altered and Delayed Inflammatory Responses to TNF-α Compared to APOE3

**Figure 5:**
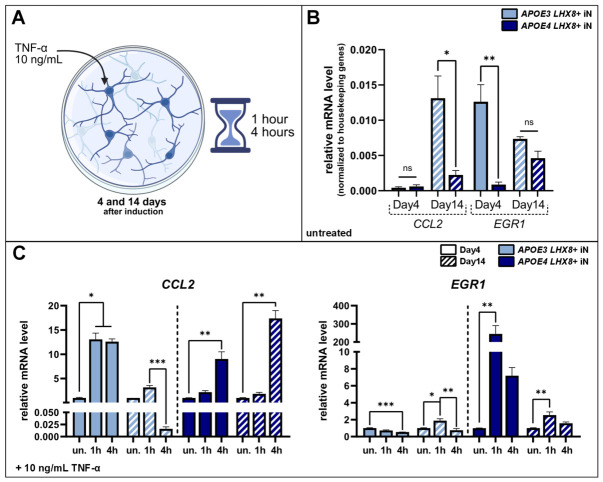
Assessment of stress and inflammatory responses following TNF-α stimulation. (**A**) Schematic illustration of the experimental design. Cholinergic-like iNs were treated with 10 ng/mL TNF-α for 1 h or 4 h. This procedure was performed at day 4 and day 14 of differentiation. (**B**) Basal expression levels of the inflammatory marker *CCL2* and the stress-associated marker *EGR1* at day 4 and day 14, comparing *APOE3* and *APOE4* iNs. (**C**) Changes in *CCL2* and *EGR1* mRNA levels in response to TNF-α stimulation. Data are presented as mean ± SEM. All samples were normalized to housekeeping genes. For basal expression (**B**), statistical analysis was performed using the Mann–Whitney test for each gene at the respective time point. For TNF-α-treated conditions (**C**), samples were analyzed relative to untreated controls (un.) from the same differentiation day using the Kruskal–Wallis test. Statistical significance was defined as * *p* < 0.05, ** *p* < 0.01, *** *p* < 0.001, while *p* > 0.05 was considered as non-significant (ns).

To mimic an inflammatory stimulus, TNF-α, which has been reported to be upregulated in tissues of AD patients and associated with lesion formation, was applied to cholinergic-like iNs carrying either an *APOE3* or *APOE4* genotype [48]. Differentiation time points at day 4 and day 14 were analyzed to investigate inflammatory responses at distinct maturation stages. In addition, iNs were examined after 1 h and 4 h of TNF-α stimulation to capture both early and adaptive responses over time (Figure 5A). To gain insight into general inflammatory response patterns, mRNA levels of *CCL2* and *EGR1* were analyzed. CCL2 is widely recognized as a mediator of inflammatory processes and is frequently upregulated under inflammatory conditions [49,50]. In contrast, EGR1 is closely linked to acute stress responses and is commonly used as a marker of neuronal activity [51]. To first assess genotype-specific baseline differences, basal expression levels were compared prior to TNF-α stimulation (Figure 5B). At day 4, *EGR1* expression was significantly higher in *APOE3* iNs, whereas *CCL2* levels did not differ significantly. At day 14, this pattern shifted: *CCL2* expression was elevated in *APOE3* iNs, while no significant differences in *EGR1* expression were observed between the genotypes. Following TNF-α stimulation (Figure 5C), *APOE3* iNs exhibited a rapid and sustained increase in *CCL2* expression, detectable as early as 1 h and maintained at 4 h. In contrast, *APOE4* iNs showed a delayed response, with a pronounced increase only after 4 h of stimulation (Figure 5C, Mean ΔCq values in Table S4). At day 14, the response pattern in *APOE3* iNs differed markedly. A slight, non-significant increase was observed after 1 h, followed by a downregulation at 4 h. In contrast, *APOE4* iNs maintained a response pattern similar to that observed at day 4, despite exhibiting higher basal *CCL2* expression levels. In contrast to *CCL2*, *EGR1* displayed a distinct regulatory pattern. At day 4, *APOE3* iNs showed a slight reduction in *EGR1* expression over the time of TNF-α stimulation. In *APOE4* iNs, however, *EGR1* expression was strongly induced (~240-fold) after 1 h of stimulation, followed by a substantial decline at 4 h. Basal *EGR1* levels were higher at day 14, but the relative induction after 1 h was markedly attenuated compared to day 4, while still exhibiting an early peak. Notably, the overall regulation pattern at day 14 resembled that of *APOE3* iNs more closely, although they showed a more pronounced reduction after 4 h of stimulation.

### 3.6. Regulation of Lipid-Related Genes in APOE4 iNs During Inflammation Is Stage-Dependent

**Figure 6:**
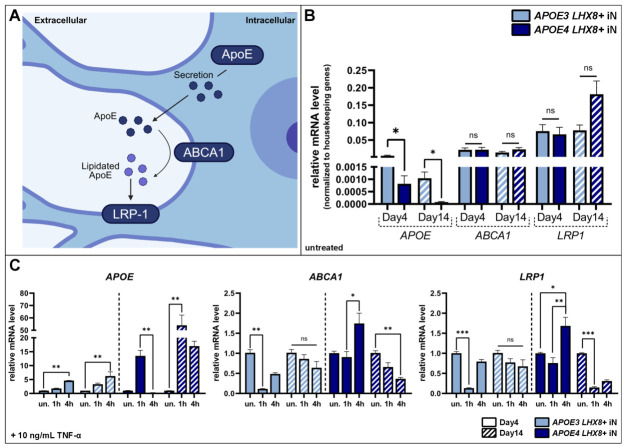
Effect of TNF-α stimulation on mRNA levels of genes involved in lipid transport. (**A**) Schematic illustration of *APOE*-related lipid transport. ApoE is produced and secreted by neurons. ABCA1 mediates the lipidation of ApoE, while LRP-1 facilitates the transport and trafficking of lipidated ApoE. (**B**) Basal mRNA levels of *APOE*, *ABCA1*, and *LRP1* at day 4 and day 14 in *APOE3* and *APOE4 LHX8*+ iNs. (**C**) The mRNA level changes in *APOE*, *ABCA1* and *LRP1* following TNF-α treatment in *APOE3* and *APOE4* iNs at day 4 and day 14, analyzed after 1 h and 4 h of stimulation. Data are presented as mean ± SEM. All samples were normalized to housekeeping genes. For basal expression (**B**), statistical analysis was performed using the Mann–Whitney test for each gene at the respective time point. For TNF-α-treated conditions (**C**), samples were analyzed relative to untreated controls (un.) from the same differentiation day using the Kruskal–Wallis test. Statistical significance was defined as * *p* < 0.05, ** *p* < 0.01, *** *p* < 0.001, while *p* > 0.05 was considered as non-significant (ns).

Given the strong link between Aβ production and impaired lipid transport, genes involved in ApoE-mediated lipid handling (*APOE*, *ABCA1* and *LRP1*) were analyzed to assess the effects of TNF-α stimulation as a model of inflammatory response (Figure 6A) [52,53,54]. At baseline, a notable difference was observed at both differentiation stages, with *APOE* mRNA levels being significantly elevated in *APOE3* iNs. In contrast, *ABCA1* and *LRP1* expression levels were roughly comparable between genotypes, although *LRP1* expression appeared elevated in *APOE4* iNs at day 14, despite not reaching statistical significance (Figure 6B). Upon TNF-α stimulation, distinct genotype-dependent responses became evident (Figure 6C, Mean ΔCq values in Table S5). In *APOE3* iNs, *APOE* expression increased progressively over treatment time at both early and later stages. In contrast, *APOE4* iNs exhibited a rapid and pronounced upregulation after 1 h of stimulation, followed by a significant downregulation at 4 h. This dynamic pattern was also observed at day 14, although the extent of change appeared more pronounced, potentially reflecting very low basal *APOE* expression levels in untreated conditions. Notably, despite the decline at 4 h, *APOE* mRNA levels remained elevated compared to the control. *ABCA1* and *LRP1* displayed more pronounced differences depending on the stage of neuronal differentiation. In *APOE3* iNs after 4 days of differentiation, both genes were downregulated after 1 h of TNF-α treatment but showed a trend toward recovery, with no significant differences remaining after 4 h. At the more mature stage, no significant changes were detected, although a slight trend toward decreased expression was observed. Conversely, *APOE4* iNs at day 4 showed an opposing response, characterized by an increase in expression after 4 h of stimulation. Interestingly, at day 14, *APOE4* iNs exhibited downregulation following TNF-α exposure, with *ABCA1* following a more continuous pattern, while *LRP1* reached its lowest expression level at 1 h.

### 3.7. APOE Genotype and Neuronal Maturation Differentially Shape Amyloidogenic Pathway Responses to TNF-α Stimulation

**Figure 7:**
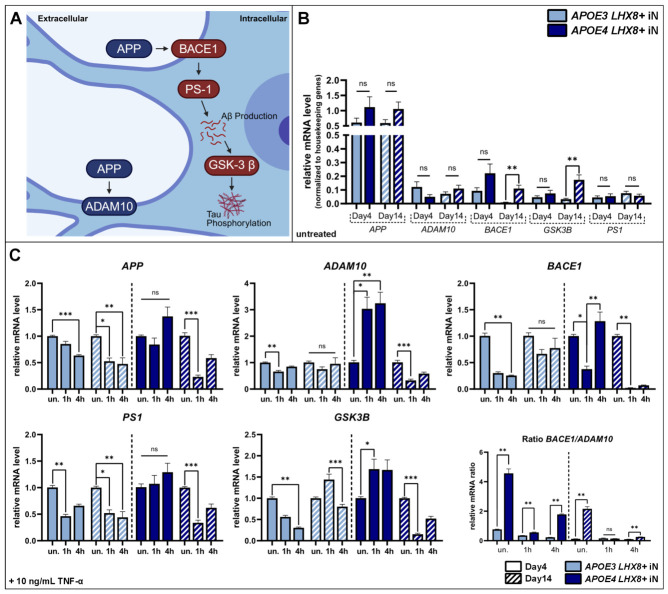
TNF-α-induced regulation of key components of the amyloidogenic pathway. (**A**) Schematic illustration of the non-amyloidogenic and amyloidogenic pathways. The amyloid precursor protein (APP) is processed either by BACE1 or ADAM10. Cleavage by ADAM10 represents the non-amyloidogenic pathway. In contrast, processing by BACE1 initiates the amyloidogenic cascade associated with AD. BACE1 cleaves APP, followed by γ-secretase processing (PS-1), generating Aβ peptides. Accumulation of Aβ can promote downstream signaling events, including activation of GSK-3 β, which contributes to Tau phosphorylation. (**B**) Basal mRNA levels of genes involved in the amyloidogenic pathway (*APP*, *ADAM10*, *BACE1*, *GSK3B*, and *PS1*) comparing *APOE3* and *APOE4* iNs at day 4 and day 14 of differentiation. (**C**) Changes in gene expression following TNF-α stimulation, as well as the *BACE1*/*ADAM10* ratio over the course of treatment. For basal expression (**B**), statistical analysis was performed using the Mann–Whitney test for each gene at the respective time point. For TNF-α-treated conditions (**C**), samples were analyzed relative to untreated controls (un.) from the same day of differentiation using the Kruskal–Wallis test. The *BACE1*/*ADAM10* ratio was calculated based on relative mRNA levels normalized to housekeeping genes. Comparisons were performed within the same condition and differentiation time point, and statistical analysis was conducted using the Mann–Whitney test. Statistical significance was defined as * *p* < 0.05, ** *p* < 0.01, *** *p* < 0.001, while *p* > 0.05 was considered as non-significant (ns).

The amyloidogenic pathway is responsible for Aβ production, which in turn contributes to tau phosphorylation and is associated with key biomarkers of Alzheimer’s disease (Figure 7A). Therefore, genes involved in this pathway were analyzed under inflammatory conditions induced by TNF-α stimulation. These include the APP-processing secretases *ADAM10* and *BACE1*, as well as *APP* itself. In addition, *PS1*, which is involved in APP cleavage, and *GSK3B*, a kinase known to promote tau phosphorylation, were examined [55]. Baseline expression levels of *APP*, *ADAM10*, *BACE1*, *GSK3B* and *PS1* in untreated iNs did not differ significantly between *APOE3* and *APOE4* genotypes at day 4 (Figure 7B). However, at day 14, significant differences became evident, particularly in *BACE1* and *GSK3B* expression. Upon TNF-α induction, genotype-specific responses emerged (Figure 7C, Mean ΔCq values in Table S6). In *APOE3* iNs, *APP* expression was significantly reduced after 4 h at day 4, whereas no significant changes were observed in *APOE4* iNs at this stage. At day 14, *APP* levels were already significantly decreased after 1 h in both genotypes. While *APP* expression remained low in *APOE3* iNs after 4 h, *APOE4* iNs showed a partial recovery, although levels remained below untreated conditions. *ADAM10*, representing the non-amyloidogenic processing pathway, showed a slight downregulation after 1 h in *APOE3* iNs, which reached significance only at day 4. In contrast, *APOE4* iNs exhibited a marked upregulation at day 4 following TNF-α stimulation, whereas at day 14, expression was reduced. The gene *BACE1* for the complementary enzyme was downregulated in *APOE3* iNs at day 4, while at day 14 only a non-significant downward trend was observed. In *APOE4* iNs, *BACE1* initially decreased after 1 h. However, at day 14, a compensatory increase was detected, with expression levels slightly exceeding control conditions at later time points. Notably, at day 4, *BACE1* expression was strongly reduced after 1 h of stimulation. Overall, the ratio between *BACE1* and *ADAM10* indicated a relative predominance of *BACE1* in *APOE4* compared to *APOE3* iNs, while the baseline ratio in untreated cells was higher at day 4 than at day 14. Extending the analysis to downstream APP processing, *PS1* expression was evaluated. Here, *PS1* expression was reduced at both day 4 and day 14, following a pattern similar to that of *APP*. Finally, *GSK3B* expression was assessed to evaluate downstream effects related to tau phosphorylation. In *APOE3* iNs at day 4, *GSK3B* showed a continuous downregulation over time, a pattern not observed in *APOE4* iNs, where expression instead increased. At day 14, *APOE3* iNs exhibited a slight transient increase after 1 h that normalized by 4 h, resulting in an overall significant reduction. In contrast, *APOE4* iNs displayed an initial downregulation after 1 h, followed by a modest recovery at 4 h. Taken together, these findings reveal not only genotype-specific differences but also clear stage-dependent responses to TNF-α stimulation. Notably, *APOE4* iNs tend to exhibit upregulation at earlier stages (day 4), whereas later stages (day 14) are characterized by a shift toward downregulation, resembling the pattern previously observed for lipid-related genes.

## 4. Discussion

Although AD remains extensively studied, effective therapeutic strategies are still lacking, with currently available treatments largely limited to symptomatic relief. The lack of effective treatment options highlights the importance of employing diverse and complementary research approaches. In addition to animal models and patient-based studies, in vitro human cell culture systems represent a valuable tool. In this study, we established a rapid and experimentally accessible differentiation approach for the generation of cholinergic-like neurons from hiPSCs. The use of hiPSCs further enables the analyses of early developmental processes in a controlled genetic background and allows direct comparison with isogenic controls.

To investigate the effects of the AD risk allele *APOE4* in cholinergic-like neurons, we compared hiPSCs carrying a homozygous *APOE4* genotype with their isogenic *APOE3* control and differentiated them via *LHX8* overexpression. Doxycycline-triggered *LHX8* expression induced a robust morphological transition toward a neuronal phenotype with typical cholinergic markers. Consistent with previously published protocols for cholinergic neuron differentiation, pronounced ganglion-like aggregates of neuronal somata accompanied by fasciculated neurite bundles became apparent during differentiation [56,57,58]. At the morphological level, both genotypes initially showed a similar neuronal appearance, whereas genotype-specific differences became apparent at later stages. Between days 7 and 14, *APOE4* iNs appeared to form denser and more compact clusters compared to *APOE3* iNs.

Overall, at protein level, neuronal differentiation appeared to be slightly accelerated in *APOE4* iNs. Specifically, the neural progenitor marker Nestin was downregulated earlier, whereas the expression of the neuronal markers Beta-3 tubulin and MAP2 was roughly comparable, with a slight increase observed. These findings are consistent with previous reports in other neuronal model systems suggesting altered or accelerated differentiation dynamics in the context of *APOE4* [19,20,21]. Importantly, this apparent early acceleration did not result in more advanced maturation. Instead, synaptophysin expression remained low at early stages and increased only at day 14, pointing to delayed synaptic development. However, since synaptophysin represents a presynaptic marker and the present study did not include functional validation, such as electrophysiological recordings or synaptic activity measurements, this finding should be interpreted as a molecular indication of delayed synaptic maturation rather than direct evidence of impaired neuronal function. Future studies will be required to determine whether these genotype-dependent differences translate into functional synaptic deficits. This interpretation is consistent with previous findings indicating that *APOE3* promotes synaptic plasticity, whereas *APOE4* exerts predominantly detrimental effects on synaptic integrity and function [22]. In addition, *APOE4* has been associated not only with impaired synaptic plasticity but also with earlier loss of mature dendritic spines [59]. Together, these findings may suggest that early developmental alterations already lay the foundation for later neuronal dysfunction. In fact, synaptic loss is one of the earliest pathological hallmarks of AD, with *APOE4* being considered a major driving factor in this process [53]. In line with this, most presynaptic cholinergic markers analyzed here, including *ChAT*, *VACHT,* and *ACHE*, exhibited elevated mRNA levels in *APOE4* iNs, whereas *CHT1* expression was increased in *APOE3* iNs. In another neuronal model, *CHT1* has been described as a marker of the final stage of cholinergic differentiation [60]. This observation is further supported by findings from AD patient brains, where high-affinity choline uptake, which is mediated by *CHT1*, is reduced, although this step is essential for ACh synthesis [61]. Thus, the analysis of cholinergic markers may further indicate a delay in synaptic maturation. Notably, the increased *ChAT* mRNA levels in *APOE4* iNs were not reflected at the protein level at any measured time points. This discrepancy may indicate that *APOE4* enhances transcription while translational efficiency or protein stability is impaired. In line with this, previous studies have suggested that deficits in translational control, for example, during APP processing, may contribute to AD progression [62]. As protein levels were only assessed for ChAT in the present study, future analyses should investigate whether similar discrepancies also occur for other cholinergic markers, such as AChE and VAChT, to determine whether this effect is ChAT-specific or reflects a broader dysregulation of transcriptional-to-translational responses during early maturation.

To investigate both the effects of *APOE4* on neuronal differentiation and stage-dependent inflammatory responses, analyses were performed at two differentiation stages. To improve readability and facilitate comparison across differentiation stages, genotypes, treatment conditions, and genes, the main RT-qPCR findings are summarized in Table A1. Given the established role of TNF-α in Alzheimer’s disease-associated neuroinflammation, stimulation was applied [6]. At day 4, representing an early differentiation stage, *APOE3* iNs showed a rapid and pronounced upregulation of *CCL2* already after 1 h of TNF-α stimulation. *CCL2* is a well-established inflammatory mediator in neuronal cells and has also been reported to be elevated in AD patients [63,64]. In contrast, *APOE4* iNs displayed a delayed response, characterized by a more gradual and continuous increase in *CCL2* expression over time. Strikingly, this genotype-specific pattern persisted and became even more pronounced at day 14. Whereas *APOE3* iNs still exhibited an initial, albeit less pronounced, increase in *CCL2* after 1 h, prolonged TNF-α stimulation resulted in a subsequent downregulation, suggesting a more adaptive or regulated response. This maturation-dependent attenuation is in line with previous reports that neuronal responses to TNF-α are age-dependent [65]. By contrast, *APOE4* iNs failed to exhibit comparable attenuation and instead maintained elevated *CCL2* levels. Supporting this, previous murine studies have demonstrated that neuronal *APOE4* expression is associated with sustained *CCL2* induction, thereby exacerbating neuroinflammatory responses [66]. Furthermore, *CCL2* has been implicated in accelerating plaque formation and contributing to cognitive decline in AD [64,67]. Thus, the sustained *CCL2* expression observed in *APOE4* iNs may reflect a failure to properly resolve inflammatory signaling. Interestingly, at day 4, the delayed *CCL2* response in APOE4 iNs was accompanied by a distinct early increase in *EGR1* expression after 1 h of TNF-α stimulation. As *EGR1* is described as rapidly induced under cellular stress conditions, this finding may indicate that *APOE4* iNs initially respond to TNF-α with a stress-associated transcriptional program rather than a canonical inflammatory response [51]. Such stress-associated *EGR1* upregulation has been linked to memory deficits in animal models [68]. Notably, this effect was no longer observed at later differentiation stages, suggesting that this early stress response is transient and dependent on neuronal maturation state. Together, these findings further support the notion that *APOE4*-associated alterations are not limited to aging neurons, but may already occur during early neuronal differentiation, where even transient stress-related responses could potentially affect subsequent developmental programs.

The maturation-dependent modulation of inflammatory signaling was also reflected in the regulation of lipid transport-related genes. ApoE is a central cholesterol and lipid transport protein in the brain and thereby contributes to neuronal lipid homeostasis, synaptic maintenance, and repair processes [10,69]. In this context, ApoE4-associated alterations may not only affect neuroinflammatory signaling but also disturb lipid transport pathways that are essential for neuronal function. In *APOE3* iNs, the effects of TNF-α on genes such as *ABCA1* and *LRP1* were more evident at day 4 than at day 14. At the early stage, a transient downregulation of these lipid metabolism-associated transcripts was observed, followed by partial recovery after 4 h of stimulation. In contrast, no significant changes were detected at day 14, consistent with the notion that more mature neurons mount a generally attenuated response to TNF-α. Given that reduced ABCA1 function has been linked to enhanced inflammatory responses in mouse models, a more tightly regulated lipid metabolism may even be beneficial in limiting excessive inflammation [70]. By contrast, *APOE4* iNs exhibited persistent regulation of lipid metabolism–related genes at both time points, in line with the sustained inflammatory response observed at day 14. This may also be associated with a more pronounced regulation of *APOE* expression itself. *APOE* expression has been reported to increase the response to cellular stress, consistent with the marked transient peak observed in *APOE4* iNs after 1 h of TNF-α stimulation [71]. Whereas *APOE3* iNs showed a more moderate and progressive increase in *APOE* expression, *APOE4* iNs displayed a sharp early peak. On day 4, this peak was followed by a reduction after prolonged stimulation, potentially reflecting a compensatory response to impaired lipid transport. However, at day 14, *APOE* expression remained elevated over time, albeit to a lesser extent, suggesting a sustained or dysregulated response to inflammatory stimulation rather than efficient resolution. In this context, this sustained *APOE* induction occurred in parallel with persistent downregulation or only partial recovery of key lipid transport mediators such as *ABCA1* and *LRP1*. This divergence may indicate that, despite increased *APOE* expression, the downstream lipid transport machinery becomes functionally impaired. Notably, *APOE4* has been reported to promote the downregulation of *LRP1* and its dynamic regulation in response to TNF-α may lead to this effect [72]. Disruption of the functional interaction between *APOE* and *LRP1* has been associated with impaired neuronal function, particularly affecting synapses [53]. Thus, the observed dysregulation of lipid metabolism in *APOE4* iNs may provide a mechanistic link to the impaired synaptic marker expression described above. Outside of the central nervous system (CNS), *APOE4* has also been associated with altered systemic lipid metabolism and dyslipidemia, emphasizing that APOE4-related disease mechanisms may involve both central neuronal lipid handling and broader disturbances in cholesterol transport [69]. However, since the present study was performed in neuronal cultures, systemic lipid metabolic effects could not be directly evaluated in this model. In addition, as *LRP1* plays a critical role in the clearance of Aβ, its downregulation under inflammatory conditions may further exacerbate neuronal vulnerability in AD [52,54]. Consistently, brain-specific *LRP1* silencing or knockdown in murine studies has been shown to aggravate AD-related neuropathology [73,74].

Given the close relationship between lipid metabolism and amyloid precursor protein processing, we next examined genes at the mRNA level involved in the amyloidogenic pathway to gain further insight into potential effects on Aβ-related mechanisms. In *APOE3* iNs, the transcriptional response of APP-processing–related genes was largely consistent across developmental stages. Overall, several genes were downregulated at the mRNA level, which may reflect a protective response aimed at limiting amyloidogenic processing under inflammatory conditions. Even the slight increase in *GSK3B* gene expression at day 14 was reversed after prolonged TNF-α stimulation. Moreover, the ratio between gene expression of *BACE1* and *ADAM10* remained relatively stable and even shifted slightly toward *ADAM10* over time, suggesting a balanced regulation that favors non-amyloidogenic processing profile at the transcriptional level. This trend was even more evident at day 14, when neither *BACE1* nor *ADAM10* showed significant changes, further supporting the notion that more mature *APOE3* iNs exhibit an attenuated transcriptional response to TNF-α. In contrast, *APOE4* iNs displayed a distinct and less controlled response pattern. At early time points, *APP* gene expression did not show clear downregulation and instead tended to increase, although not significantly. While *ADAM10* was upregulated and *BACE1* initially downregulated, suggesting a transient protective response, the overall ratio remained shifted toward *BACE1* compared to *APOE3* iNs. Importantly, this initial transcriptional downregulation of *BACE1* was not sustained, as its mRNA levels increased again after prolonged TNF-α stimulation. This pattern suggests an unstable regulatory response in *APOE4* iNs, characterized by transient compensation followed by loss of control under sustained inflammatory conditions. In addition, the increase in *GSK3B* expression observed at day 4 in *APOE4* iNs, in contrast to its reduction in *APOE3* iNs, may contribute to the altered regulation on mRNA level of lipid metabolism–related genes such as *LRP1* and *ABCA1*, as *GSK3B* has been described as a regulator of lipid homeostasis [75]. Interestingly, at day 14, a general downregulation of APP-processing–related genes was observed in *APOE4* iNs. Although this may appear counterintuitive, given the established role of *APOE4* in promoting amyloidogenic pathways, may reflect an early adaptive or stress-induced transcriptional suppression in response to inflammatory challenge [76]. Notably, the strongest effect was observed after 1 h of stimulation, suggesting a transient suppression that may not be maintained during prolonged inflammatory exposure. It remains possible that chronic neuroinflammatory conditions would ultimately lead to reactivation, rather than suppression, of amyloidogenic pathways. However, it is important to consider that GSK-3 β is primarily regulated at the level of its activation rather than expression [77]. Since this study assessed only the transcriptional level and not the translational level, potential changes at the protein level may differ from the observed gene expression patterns. Future studies assessing GSK-3 β protein abundance, phosphorylation status, and kinase activity would therefore be important to clarify whether the observed transcriptional changes translate into functional alterations of GSK-3 β signaling. Furthermore, it is important to note that the present study focused on neuronal cultures, whereas ApoE-related inflammatory effects have been strongly associated with glial cells, including astrocytes [78]. Astrocytes are considered the major source of ApoE in the brain and contribute substantially to extracellular/secreted ApoE, while neuronal ApoE expression is generally lower and may be particularly relevant under stress or injury conditions [6,79]. Therefore, the absence of astrocytes in the present culture system represents an important limitation, as astrocyte-derived ApoE and glia-mediated neuron–glia interactions are not captured. Therefore, the observed neuronal response may only partially reflect the complexity of *APOE4*-associated neuroinflammatory processes in vivo. At the same time, this neuron-enriched culture system allows the investigation of neuron-intrinsic, cell-autonomous *APOE4* effects without additional modulation by glial-derived signals. However, future studies incorporating astrocytes or other glial cells in co-culture systems will be necessary to assess how neuron–glia interactions influence the ApoE4-associated responses observed in the present study.

Our study demonstrates that the detrimental effects of *APOE4* on neuronal function are not restricted to advanced stages of disease but emerge already during early neuronal differentiation. Most notably, synaptogenesis appeared to be delayed in *APOE4* iNs, indicating early impairments in neuronal maturation. In addition, inflammatory responses were differentially regulated between genotype-dependent manner. At the early stage of differentiation, both genotypes responded transcriptionally to TNF-α, but *APOE4* iNs showed an additional prominent stress-associated component. As differentiation progressed, *APOE3* iNs displayed a reduced and more tightly controlled inflammatory response, suggestive of adaptive regulation. In contrast, *APOE4* iNs failed to attenuate this response and instead maintained a heightened inflammatory state. Such persistent activation may promote progressive cellular dysfunction and, under sustained inflammatory conditions, may ultimately culminate in a broader suppression of neuronal homeostatic programs. A major limitation of the present study is that the experiments were performed using one hiPSC line per genotype. Thus, although the use of isogenic *APOE3*/*APOE4* lines allows the analysis of *APOE* genotype-associated effects in a controlled genetic background, the absence of additional independent biological replicates limits the generalizability of the findings. Therefore, the observed ApoE4-associated alterations should be interpreted with caution and regarded as hypothesis-generating. Future studies including multiple independently derived hiPSC lines per genotype will be necessary to validate whether these effects persist across different genetic backgrounds. Furthermore, the present study focuses on an early developmental window of TF-mediated neuronal differentiation, covering days 4 to 14 in vitro. This period likely reflects early stages of neuronal lineage commitment and initial neuronal maturation rather than fully mature or aged neuronal states. In line with previous studies on directed neuronal differentiation, forced expression of proneural transcription factors activates transcriptional cascades that overlap with early in vivo neurodevelopmental programs, including factors involved in neuronal fate specification and early neural differentiation [80]. Therefore, the observed *APOE4*-associated changes should be interpreted as early developmental alterations in differentiating iNs. Since AD is primarily an age-associated neurodegenerative disease, *APOE4*-related effects in mature or aged neurons may differ from the early alterations observed here and could involve additional mechanisms such as accumulated cellular stress, altered proteostasis, mitochondrial dysfunction, or age-dependent changes in lipid metabolism. Thus, longer differentiation periods or aging-associated experimental paradigms will be required to determine whether these effects persist, normalize, or further diverge during later neuronal maturation and aging. Consistent with previous reports demonstrating that *APOE4*-associated alterations arise already during neurogenesis, the persistence of the changes observed in this study throughout early neuronal development is not unexpected [21]. These findings support the notion that *APOE4* contributes early on to the establishment of a cellular environment predisposed to Alzheimer’s disease. Importantly, this highlights a temporal shift in disease vulnerability, suggesting that key pathogenic processes may be initiated long before clinical symptoms become apparent. Therefore, a deeper focus on early developmental stages will be essential to fully understand AD pathogenesis, as molecular and cellular dysfunctions are likely initiated well before the onset of overt neurodegeneration.

## 5. Conclusions

This study investigated the impact of a homozygous *APOE4* background on the development of human cholinergic-like neurons. For this purpose, we established a rapid differentiation model based on *LHX8* overexpression, enabling the generation of iNs with cholinergic marker expression from hiPSCs. This model demonstrated that *APOE4* is associated with delayed synaptic maturation during neuronal differentiation. Moreover, *APOE4* iNs displayed stage-dependent alterations in their response to TNF-α stimulation. In contrast to *APOE3* iNs, *APOE4* iNs did not develop a comparable adaptive inflammatory response over the same differentiation period. Together, these results support the concept that *APOE4*-related alterations are not restricted to aged neurons but may already arise during early neuronal development, thereby contributing to a cellular environment that is more vulnerable to AD-associated stressors.

## Figures and Tables

**Table 1:** Ratio of E8 Flex medium to neuronal differentiation medium during the first 5 days of neuronal differentiation.

Day	E8 Flex Medium	Neuronal Differentiation Medium
0	100%	0%
1	75%	25%
2	50%	50%
3	50%	50%
4	25%	75%
5	0%	100%

**Table 2:** Overview of primary antibodies and their dilutions used in this study.

Primary Antibody	Host	Dilution	Manufacturer
Beta-3 tubulin	mouse	1:2000	Promega GmbH, Walldorf, Germany
ChAT	rabbit	1:400	Sigma Aldrich, St. Louis, MO, USA
MAP2	mouse	1:200	Thermo Fisher Scientific, Waltham, MA, USA
Nestin	mouse	1:200	Merck Millipore, Burlington, MA, USA
Oct-4A	rabbit	1:200	Cell Signaling Technology, Danvers & Boston, MA, USA
SSEA4	mouse	1:200	Cell Signaling Technology, Danvers & Boston, MA, USA
Synaptophysin	rabbit	1:500	Abcam, Cambridge, UK
vGlut2	mouse	1:200	Merck Millipore, Burlington, MA, USA

## Data Availability

The original contributions presented in this study are included in the article/Supplementary Material. Further inquiries can be directed to the corresponding author.

## Supplementary Materials

The following supporting information can be downloaded at: https://www.mdpi.com/article/10.3390/cells15121057/s1, Figure S1: Exclusion of a glutamatergic phenotype in LHX8-induced iNs; Table S1: List of primers used for RT-PCR and qPCR; Table S2: Quantification of ICC fluorescence intensities in *APOE3* and *APOE4* iNs during differentiation; Table S3: Mean ΔCq values of cholinergic marker genes used for relative mRNA quantification in Figure 4; Table S4: Mean ΔCq values of *CCL2* and *EGR1* used for relative mRNA quantification; Table S5: Mean ΔCq values of *APOE*, *ABCA1* and *LRP1* used for relative mRNA quantification; Table S6: Mean ΔCq values of *APP*, *ADAM10*, *BACE1*, *PS1* and *GSK3B* used for relative mRNA quantification.

## Abbreviations

The following abbreviations are used in this manuscript:

AD	Alzheimer’s Disease
Aβ	Amyloid-β
EOAD	Early-onset Alzheimer’s Disease
LOAD	Late-onset Alzheimer’s Disease
APOE	Apolipoprotein E
ACh	Acetylcholine
TF	Transcription factor
iN	Induced Neurons
TNF-α	Tumor necrosis factor alpha
hiPSCs	Human-induced pluripotent stem cell
ChAT	Choline Acetyltransferase
VAChT	Vesicular acetylcholine transporter
AChE	Acetylcholinesterase
CHT1	High affinity choline transporter 1
CCL2	C-C motif chemokine ligand 2
EGR1	Early Growth Response 1
ABCA1	ATP-Binding Cassette Transporter A1
LRP-1	Low Density Lipoprotein Receptor-related Protein 1
APP	Amyloid-beta precursor protein
ADAM10	Disintegrin and metalloproteinase domain-containing protein 10
BACE1	Beta-Site APP-Cleaving Enzyme 1
PS1	Presenilin-1
GSK3B	Glycogen synthase kinase-3 beta

## Appendix A

**Table A1:** Summary of RT-qPCR results stratified by differentiation stage, genotype, and gene. Fold changes are shown for each treatment time point relative to the untreated control (un.). The samples were analyzed using the Kruskal–Wallis test. Statistical significance was defined as * *p* < 0.05, ** *p* < 0.01, *** *p* < 0.001, while *p* > 0.05 was considered as non-significant (ns).

Differentiation Stage	Genotype	Gene	Treatment Response			
			1h TNF-α vs. un.		4h TNF-α vs. un.	
Day 4	APOE3	*CCL2*	13.0×	↑ *	12.6×	↑ *
Day 14			3.2×	ns	0.02×	ns
Day 4	APOE4		2.2×	ns	9×	↑ **
Day 14			1.9×	ns	17.4×	↑ **
Day 4	APOE3	*EGR1*	0.7×	ns	0.5×	↓ ***
Day 14			1.9×	↑ *	0.8×	ns
Day 4	APOE4		244.5×	↑ **	7.2×	ns
Day 14			2.5×	↑ **	1.6×	ns
Day 4	APOE3	*APOE*	1.8×	ns	4.6×	↑ **
Day 14			3.3×	ns	6.3×	↑ **
Day 4	APOE4		13.5×	ns	0.3×	ns
Day 14			54.1×	↑ **	17.0×	ns
Day 4	APOE3	*ABCA1*	0.1×	↓ **	0.5×	ns
Day 14			0.9×	ns	0.6×	ns
Day 4	APOE4		0.9×	ns	1.7×	ns
Day 14			0.7×	ns	0.4×	↓ **
Day 4	APOE3	*LRP1*	0.1×	↓ ***	0.8×	ns
Day 14			0.8×	ns	0.7×	ns
Day 4	APOE4		0.8×	ns	1.7×	↑ *
Day 14			0.1×	↓ ***	0.3×	ns
Day 4	APOE3	*APP*	0.9×	ns	0.6×	↓ ***
Day 14			0.5×	↓ *	0.5×	↓ **
Day 4	APOE4		0.8×	ns	1.4×	ns
Day 14			0.2×	↓ ***	0.6×	ns
Day 4	APOE3	*ADAM10*	0.7×	↓**	0.8×	ns
Day 14			0.8×	ns	1×	ns
Day 4	APOE4		3×	↑ *	3.2×	↑ **
Day 14			0.3×	↓ ***	0.6×	ns
Day 4	APOE3	*BACE1*	0.3×	ns	0.3×	↓ **
Day 14			0.7×	ns	0.8×	ns
Day 4	APOE4		0.4×	↓ *	1.3×	ns
Day 14			0.02×	↓ **	0.07×	ns
Day 4	APOE3	*PS1*	0.5×	↓ **	0.7×	ns
Day 14			0.5×	↓ *	0.4×	↓ **
Day 4	APOE4		1×	ns	1.3×	ns
Day 14			0.3×	↓ ***	0.6×	ns
Day 4	APOE3	*GSK3B*	0.6×	ns	0.3×	↓ **
Day 14			0.9×	ns	0.6×	ns
Day 4	APOE4		1.7×	↑ *	1.7×	ns
Day 14			0.2×	↓ ***	0.5×	ns
