# Supplementary material for: Cholinergic Differentiation of Human iPSCs Reveals Early APOE4-Driven Dysregulation of Neuronal Markers, Synaptogenesis and Inflammatory Responses
Source: Cells. 2026 Jun 9;15(12):1057. doi: 10.3390/cells15121057 (PMC13296407; doi:10.3390/cells15121057)
Supplement: Supplementary file 1 [file cells-15-01057-s001.zip › Supplementary Figures.pdf]

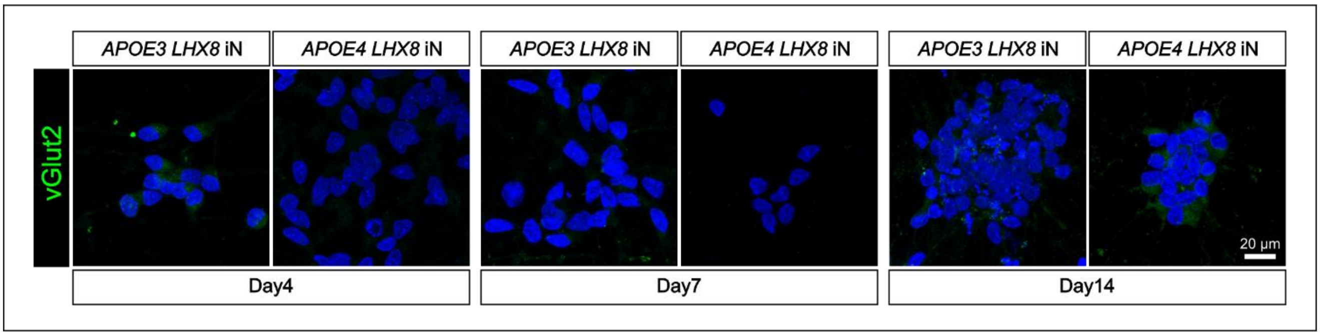

Figure S1: ICC detection of vGlut2 in APOE3 and APOE4 iNs generated via LHX8-mediated differentiation, performed to exclude a glutamatergic lineage at days 4, 7, and 14. The lack of vGlut2 protein expression suggests a non-glutamatergic phenotype. Cell nuclei were counterstained with DAPI (blue).
