# Supplementary material for: Cholinergic Differentiation of Human iPSCs Reveals Early APOE4-Driven Dysregulation of Neuronal Markers, Synaptogenesis and Inflammatory Responses
Source: Cells. 2026 Jun 9;15(12):1057. doi: 10.3390/cells15121057 (PMC13296407; doi:10.3390/cells15121057)
Supplement: Supplementary file 1 [file cells-15-01057-s001.zip › Supplementary Tables_ProofRead.pdf]

Table S1: List of primers used for RT-PCR and qPCR.

Primer	Sequence 5′→ 3′
Plasmid-derived *LHX8* fwd	TCACTTGGCATGCTTTGCCTGC
Plasmid-derived *LHX8* rev	CGCACCGTGGGCTTGTACTCGG
ABCA1 fw	CAGGCTACTACCTGACCTTGGT
ABCA1 rev	CTGCTCTGAGAAACACTGTCCTC
ACHE fw	GTTCTCCTTCGTGCCTGTGGTA
ACHE rev	ATACGAGCCCTCATCCTTCACC
ADAM10 fw	GAGGAGTGTACGTGTGCCAGTT
ADAM10 rev	GACCACTGAAGTGCCTACTCCA
APOE fw	GGGTCGCTTTTGGGATTACCTG
APOE rev	CAACTCCTTCATGGTCTCGTCC
APP fw	CCTTCTCGTTCCTGACAAGTGC
APP rev	GGCAGCAACATGCCGTAGTCAT
BACE1 fw	GTGAGGTTACCAACCAGTCCTTC
BACE1 rev	CGTGGATGACTGTGAGATGGCA
CCL2 fw	CAGCCAGATGCAATCAATGCC
CCL2 rev	TGGAATCCTGAACCCACTTCT
CHAT fw	GTGGCTCAGAACAGCAGCATCA
CHAT rev	CCTCACTGAGACGGCGGAAATT
CHT1 fw	AAAAGCCGTGGCTGGGAACTGT
CHT1 rev	GCACTTGAGCATAGGTGGCTGA
EGR1 fw	AGAAGGACAAGAAAGCAGACAAAAGTGT
EGR1 rev	GGGGACGGGTAGGAAGAGAG
GAPDH fw	CATGAGAAGTATGACAACAGCCT
GAPDH rev	AGTCCTTCCACGATACCAAAGT
GSK3B fw	CCGACTAACACCACTGGAAGCT
GSK3B rev	AGGATGGTAGCCAGAGGTGGAT
LRP1 fw	CAACGGCATCTCAGTGGACTAC
LRP1 rev	TGTTGCTGGACAGAACCACCTC
PS1 fw	GCAGTATCCTCGCTGGTGAAGA
PS1 rev	CAGGCTATGGTTGTGTTCCAGTC
RPLP0 fw	TGGTCATCCAGCAGGTGTTCGA
RPLP0 rev	ACAGACACTGGCAACATTGCGG
VACHT fw	GCTGTTTGCTTCCAAGGCTATCC
VACHT rev	GAAGGCGAACAGGACTGTAGAG

Table S2: Quantification of ICC fluorescence intensities in *APOE3* and *APOE4* iNs during differentiation. Relative fluorescence intensities of Beta-3 tubulin, Nestin, MAP2, Synaptophysin, and ChAT were quantified at day 4, day 7, and day 14 of differentiation and are shown as mean fluorescence values and corresponding z-scores. Quantification was based on ICC staining using three microscopic images per condition from one cell line per genotype. Statistical comparisons between *APOE3* and *APOE4* iNs were performed using the Mann–Whitney test. No statistically significant genotype-dependent differences were detected at the analyzed time points ($p > 0.05$).

Genotype	Differentiation stage	Protein	z-score	Mean Fluorescence
APOE3	Day4	Beta-3 Tubulin	-0.4	373063
APOE4			-0.3	407271
APOE3	Day7		-0.4	367108
APOE4			1.6	1272831
APOE3	Day14		-0.6	254969
APOE4			0.2	537355
APOE3	Day4	Nestin	1.7	441578
APOE4			0	207173
APOE3	Day7		-0.2	189419
APOE4			-0.7	125246
APOE3	Day14		-0.5	148254
APOE4			-0.4	160788
APOE3	Day4	MAP2	-1.3	115490
APOE4			-0.9	146428
APOE3	Day7		0.2	228406
APOE4			-0.7	240935
APOE3	Day14		1	290763
APOE4			-0.4	255338
APOE3	Day4	Synaptophysin	0.4	178562
APOE4			-1.2	22491
APOE3	Day7		0	148265
APOE4			-1.1	30897
APOE3	Day14		0.3	171667
APOE4			1.4	283535
APOE3	Day4	ChAT	1.6	413380
APOE4			0.7	65033
APOE3	Day7		-0.8	62065
APOE4			-0.3	121107
APOE3	Day14		-0.7	76418
APOE4			0.8	295876

Table S3: Mean ΔCq values of cholinergic marker genes used for relative mRNA quantification in Figure 4.

Condition	ChAT	ACHE	VACHT	CHT1
APOE3 iN Day4	8.7	7.6	5.9	8.7
APOE3 iN Day7	11.5	9.2	6.6	8.0
APOE3 iN Day14	9.5	10.3	7.3	8.2
APOE4 iN Day4	7.3	5.5	4.0	11.3
APOE4 iN Day7	6.7	6.3	4.3	10.7
APOE4 iN Day14	7.5	5.2	4.2	9.1

Table S4: Mean ΔCq values of *CCL2* and *EGR1* used for relative mRNA quantification.

Differentiation stage	Genotype	Gene	Treatment		
			untreated	1h TNF-α	4h TNF-α
Day 4	APOE3	*CCL2*	11.5	7.8	7.8
	APOE4		11.1	10.1	8.0
Day 14	APOE4		6.4	4.8	12.6
	APOE4		9.0	8.2	4.9
Day 4	APOE3	*EGR1*	6.9	7.4	7.8
	APOE4		10.6	2.7	7.8
Day 14	APOE4		6.5	5.6	7.1
	APOE4		7.9	6.7	7.3

Table S5: Mean ΔCq values of *APOE*, *ABCA1* and *LRP1* used for relative mRNA quantification.

Differentiation stage	Genotype	Gene	Treatment		
			untreated	1h TNF-α	4h TNF-α
Day 4	APOE3	*APOE*	7.9	7.1	5.7
	APOE4		10.7	7.0	12.8
Day 14	APOE4		10.0	8.4	7.6
	APOE4		13.8	8.1	9.7
Day 4	APOE3	*ABCA1*	4.7	8.9	6.8
	APOE4		6.0	6.2	5.2
Day 14	APOE4		6.3	6.6	7.2
	APOE4		5.6	6.3	7.1
Day 4	APOE3	*LRP1*	4.0	6.9	4.3
	APOE4		4.4	4.3	3.7
Day 14	APOE4		3.8	4.3	4.7
	APOE4		2.7	5.5	4.4

Table S6: Mean ΔCq values of *APP*, *ADAM10*, *BACE1*, *PS1* and *GSK3B* used for relative mRNA quantification.

Differentiation stage	Genotype	Gene	Treatment		
			untreated	1h TNF-α	4h TNF-α
Day 4	APOE3	*APP*	1.0	1.2	1.6
	APOE4		0.3	0.6	-0.1
Day 14	APOE4		0.9	1.9	2.2
	APOE4		0.1	2.3	0.9
Day 4	APOE3	*ADAM10*	3.3	3.9	3.5
	APOE4		4.8	3.3	3.2
Day 14	APOE4		3.9	4.4	4.3
	APOE4		3.4	5.1	4.2
Day 4	APOE3	*BACE1*	3.7	5.4	5.6
	APOE4		2.6	4.1	2.3
Day 14	APOE4		7.0	7.6	7.6
	APOE4		2.3	7.9	6.1
Day 4	APOE3	*PS1*	4.7	5.8	5.3
	APOE4		4.6	4.6	4.3
Day 14	APOE4		3.8	4.9	5.3
	APOE4		4.3	6.0	5.0
Day 4	APOE3	*GSK3B*	4.7	5.5	6.4
	APOE4		4.2	3.5	3.5
Day 14	APOE4		5.1	5.2	6.1
	APOE4		2.7	5.5	3.7
